# Curcumin-piperine nanoparticles mitigate cuprizone-induced cognitive impairment via antioxidant and anti-inflammatory mechanisms

**DOI:** 10.3389/fnut.2025.1633855

**Published:** 2025-10-14

**Authors:** Mohammad Zubair Alam, Hala Abubaker Bagabir, Mohammad Alameen Faisal Zaher, Thamer M. A. Alqurashi, Badrah S. Alghamdi, Mohsin Kazi, Mohd Suhail, Gadah Ali Alshahrany, Noor Ahmed Alzahrani, Rafal Mohammed Bakhalgi, Mona Al-Thepyani, Hanin Abdulbaset AboTaleb, Rahaf Saeed Aldhahri, Juweiriya, Reham Tash, Ghulam Md Ashraf

**Affiliations:** ^1^Neuroscience and Geroscience Research Unit, King Fahd Medical Research Center, King Abdulaziz University, Jeddah, Saudi Arabia; ^2^Department of Medical Laboratory Sciences, Faculty of Applied Medical Sciences, King Abdulaziz University, Jeddah, Saudi Arabia; ^3^Department of Physiology, Faculty of Medicine, King Abdulaziz University, Rabigh, Rabigh, Saudi Arabia; ^4^Department of Physiology, Faculty of Medicine, King Abdulaziz University, Jeddah, Saudi Arabia; ^5^Department of Pharmacology, Faculty of Medicine, King Abdulaziz University, Rabigh, Rabigh, Saudi Arabia; ^6^Department of Physiology, Neuroscience Unit, Faculty of Medicine, King Abdulaziz University, Jeddah, Saudi Arabia; ^7^Department of Pharmaceutics, College of Pharmacy, King Saud University, Riyadh, Saudi Arabia; ^8^Department of Biochemistry, Faculty of Sciences, King Abdulaziz University, Jeddah, Saudi Arabia; ^9^Department of Microbiology, Faculty of Science, King Abdulaziz University, Jeddah, Saudi Arabia; ^10^Department of Chemistry, College of Sciences and Arts, King Abdulaziz University, Rabigh, Rabigh, Saudi Arabia; ^11^Department of Biological Sciences, Faculty of Sciences, University of Jeddah, Jeddah, Saudi Arabia; ^12^Department of Chemistry, Aligarh Muslim University, Aligarh, India; ^13^Department of Anatomy, Faculty of Medicine, King Abdulaziz University, Rabigh, Saudi Arabia; ^14^Department of Anatomy, Faculty of Medicine, Ain Shams University, Cairo, Egypt; ^15^Department of Biomedical Sciences, College of Medicine, Gulf Medical University, Ajman, United Arab Emirates

**Keywords:** inflammation, GFAP, cuprizone, cytokines, neurotoxicity

## Abstract

**Objective:**

Neuroinflammation is a key contributor to many neurodegenerative diseases. Cuprizone, a copper-chelating agent, is widely used in research to induce neurotoxicity and demyelination, mimicking the pathology of multiple sclerosis. This study investigates the protective and therapeutic effects of curcumin and piperine nanoformulations prepared in *Zanthoxylum rhetsa* seed oil against cuprizone-induced neurotoxicity in mice.

**Methods:**

Seventy-five Swiss albino mice were divided into five groups: control, cuprizone-treated, blank formulation-treated, curcumin-treated, and curcumin with piperine-treated groups. Behavioral assessments, along with biochemical and histological analyses of the hippocampus, were conducted to evaluate learning and memory, antioxidant enzyme activity, neuroinflammatory markers, and cellular integrity.

**Results:**

Cuprizone exposure significantly impaired cognitive function and induced oxidative stress, as evidenced by decreased levels of key antioxidant enzymes like catalase, superoxide dismutase, glutathione and glutathione peroxidases. Additionally, increased levels of neuroinflammatory markers such as GFAP, MCP-1, MIP-1, and CCL-5 were observed. Treatment with curcumin and piperine nanoformulations mitigated these effects by restoring antioxidant defenses and modulating inflammatory responses. The curcumin-piperine combination exhibited superior neuroprotection compared to curcumin alone, enhancing memory performance and reducing neuroinflammation more effectively. The results highlight the potential of curcumin and piperine nanoformulations in alleviating neurotoxicity and cognitive impairments associated with neurodegenerative disorders.

**Conclusion:**

These findings suggest that curcumin-based nanoformulations could serve as promising therapeutic agents for treating neuroinflammatory diseases, warranting further studies to explore their precise mechanisms and optimize their clinical applications.

## Graphical Abstract

**Figure:**
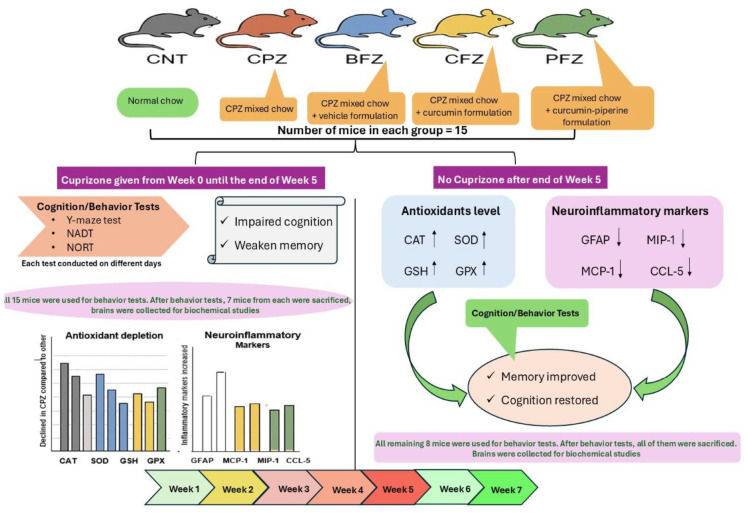
Graphical representation of the study.

## 1 Introduction

Inflammation is a highly regulated biological process characterized by the activation of immune cells and the release of signaling molecules that shape the surrounding microenvironment (1). Within the central nervous system (CNS), this process termed neuroinflammation has emerged as a key mechanism driving the onset and progression of neurological disorders. While acute inflammation plays a protective role by facilitating tissue repair and pathogen clearance, persistent or chronic neuroinflammation exerts detrimental effects, including tissue damage, synaptic dysfunction, demyelination, and neuronal death (2). Dysregulated immune responses in the CNS have been implicated not only in classical inflammatory conditions such as multiple sclerosis (MS) and vasculitis but also in neurodegenerative and cerebrovascular disorders, including Alzheimer’s disease, Parkinson’s disease, amyotrophic lateral sclerosis, stroke, and traumatic brain injury (3, 4). These pathologies are consistently associated with excessive production of pro-inflammatory cytokines, chemokines, and inducible enzymes that perpetuate neuronal injury (5).

To explore therapeutic approaches targeting neuroinflammation, the cuprizone-induced demyelination model has been widely employed. Cuprizone, a copper-chelating agent, interferes with copper-dependent enzymes essential for energy metabolism and antioxidant defense, thereby generating oxidative stress and cellular damage within brain tissue (6). It activates astrocytes and microglia, triggering a cascade of neuroinflammatory responses that contribute to neuronal degeneration (7). Furthermore, cuprizone administration disrupts antioxidant enzyme systems by reducing superoxide dismutase (SOD) and glutathione peroxidase (GPX) activity, while increasing harmful metabolites such as hydrogen peroxide and malondialdehyde (8). Since the endogenous antioxidant defense system including SOD, catalase (CAT), reduced glutathione (GSH), and GPX–plays a critical role in maintaining redox balance (9–11), cuprizone-induced deficits provide a robust platform to test antioxidant-based interventions.

Importantly, cuprizone toxicity is well-documented to alter hippocampal structure and function, leading to deficits in spatial learning, recognition memory, and cognitive flexibility (12). Given that the hippocampus governs these higher-order processes, behavioral assessments serve as functional readouts of neurodegeneration and therapeutic efficacy. In our study, the Y-maze task was utilized to evaluate spatial working memory and spontaneous alternation behavior, both sensitive markers of hippocampal dysfunction. The Novel Object Recognition Test (NORT) was employed to assess recognition memory, which depends on hippocampal–perirhinal connectivity. In addition, the Novel Arm Discrimination Test (NADT) was incorporated to examine spatial recognition and memory consolidation, providing a complementary measure of hippocampal integrity. Collectively, these behavioral paradigms validated the cognitive impairments induced by cuprizone and allowed assessment of how curcumin–piperine nanoparticles may restore hippocampal-dependent functions.

Among naturally occurring compounds, curcumin, the principal bioactive component of *Curcuma longa* (turmeric), exhibits strong neuroprotective effects due to its antioxidant and anti-inflammatory activities. Mechanistically, curcumin scavenges reactive oxygen species (ROS), inhibits the NF-κB pathway, and modulates cytokine production and other inflammatory signaling cascades (13). However, its clinical utility is limited by poor oral bioavailability. Piperine, an alkaloid from *Piper nigrum*, enhances curcumin bioavailability by up to 2000% through inhibition of glucuronidation and improvement of intestinal absorption (14). Beyond its role as a bioenhancer, piperine itself demonstrates antioxidant and anti-inflammatory effects by modulating oxidative stress markers and inflammatory mediators (15, 16).

In addition to curcumin and piperine, *Zanthoxylum rhetsa* (prickly ash or toothache tree) offers further therapeutic potential. Traditionally used in Asian medicine to alleviate digestive and inflammatory ailments, its seed oil contains essential fatty acids such as oleic and linoleic acid, along with antioxidant compounds that counteract oxidative stress (17). Historical applications for pain and swelling suggest that *Z. rhetsa* oil harbors bioactive molecules relevant in neuroinflammatory contexts (18). The combined use of curcumin, piperine, and *Z. rhetsa* seed oil represents a multi-targeted therapeutic strategy against neuroinflammation and oxidative injury. Their synergistic effects–antioxidant activity, anti-inflammatory modulation, and bioavailability enhancement are particularly suited to CNS conditions characterized by chronic inflammation. Moreover, the incorporation of these compounds into nanoparticle-based delivery systems offers a promising means to improve their pharmacokinetic profile and maximize neuroprotective efficacy in models of neurodegeneration.

Finally, we examined a range of biochemical and inflammatory markers to substantiate our findings. Glial fibrillary acidic protein (GFAP), expressed primarily in astrocytes, is critical for maintaining cellular integrity. Elevated GFAP levels accompany astrocyte activation during CNS injury, where they may aid repair but also contribute to pathology when excessively upregulated (19, 20). Chemokines such as monocyte chemoattractant protein-1 (MCP-1/CCL2) and macrophage inflammatory protein-1 (MIP-1/CCL3) are key mediators of immune cell recruitment during inflammation and are often elevated in neuroinflammatory states (21). Additionally, CCL5 (RANTES) has been reported to exert protective roles after neuronal damage by activating GPX, supporting neuronal survival under energy stress, and promoting axonal and synaptic regeneration (22, 23). In line with this, we assessed antioxidant enzymes (SOD, CAT, GPX, GSH), glial markers (GFAP), and chemokines (MCP-1, MIP-1, CCL5), alongside behavioral and histological evaluations, to provide an integrated perspective on the neuroprotective efficacy of curcumin–piperine nanoparticles in the cuprizone model.

## 2 Materials and methods

### 2.1 Animals

Seventy-five Swiss albino male mice (SWR/J) weighing between 21 and 25 g were acquired from the King Fahd Medical Research Center (KFMRC) animal housing unit at King Abdulaziz University in Jeddah, Saudi Arabia. A 12-h light/dark cycle, with the light cycle occurring between 7:00 am and 7:00 pm, was implemented to maintain mice at a suitable room temperature of 23 °C ± 2 °C and humidity level of 65%. Food and water were freely available to every mouse. Animal studies were carried out in compliance with KFMRC’s animal unit committee rules. The biomedical ethics research committee at King Abdulaziz University authorized the study protocol (approval No. ACUC-22-1-2 dated April 13, 2022), which complied with the guidelines established by the KFMRC’s Animal Care and Use Committee. The research adhered to the “System of Ethics.” The study was authorized by Royal Decree No. M/59 dated August 24, 2010, and it conformed with the “System of Ethics of Research on Living Creatures” rules created by King Abdulaziz City for Science and Technology. From June 1, 2022, until July 16, 2022, the study was conducted at the King Fahad Medical Research Center in Jeddah, Saudi Arabia. All animal experiments were designed and reported in accordance with the ARRIVE 2.0 guidelines (Animal Research: Reporting of *In Vivo* Experiments) as recommended by the EQUATOR network.^1^ This ensures transparency, reproducibility, and compliance with international ethical standards.

Only male Swiss albino mice were used in this study to reduce variability associated with hormonal fluctuations during the estrous cycle, which can influence neuroinflammatory and behavioral outcomes. This choice ensured internal consistency and reproducibility in the context of the cuprizone model.

### 2.2 Cuprizone and nanoformulations

Mice were administered 0.2% w/w cuprizone (CPZ) (purchased from Sigma-Aldrich, Bangalore, India) mixed with ground rodent chow for 5 weeks to induce acute demyelination, as previously described (24). A curcumin nanoformulation, with and without piperine, was developed by dissolving it in *Z. rhetsa* Seed Oil (ZRO), following previously published methods (25). The nanoformulations were prepared by combining various ratios of surfactant, cosurfactant, and/or co-solvent with ZRO. A fixed amount of cosurfactant (I988) and Tween 85 was used to examine the effect on formulation performance. The mixture was homogenized and stored for later use. This formulation is essentially a self-nanoemulsifying drug delivery system (SNEDDS). The SNEDDS solubility values for curcumin and piperine were 19.0 mg/g and 48.22 mg/g, respectively. The drug-free SNEDDS had a droplet size of 607.1 nm, while the curcumin-piperine loaded SNEDDS measured 700.86 nm. Zeta potentials of the curcumin-piperine loaded SNEDDS (−36.35 mV) and drug-free SNEDDS (−36.9 mV) indicated good physical stability of the resulting emulsion. The zeta potential remained consistent after curcumin-piperine loading. Curcumin was administered at 10 mg/kg, a dosage based on previous research (26). The blank and curcumin-piperine nanoformulations were diluted in normal saline to achieve concentrations of 3 mg/kg piperine and 10 mg/kg curcumin per 0.1 ml for each mouse. Daily intraperitoneal injections of 0.1 ml of the formulation were administered to each mouse between 11:00 a.m. and 1:00 p.m. Dosages were adjusted weekly based on the mice’s weight measurements.

### 2.3 Experimental design

The study lasted for 7 weeks in total in which cuprizone administered mixed with rodent chow for first 5 weeks to all groups except control. After week 5, the cuprizone was removed from diet and lasted for next 2 weeks (week 6 and 7). A total of fifteen mice each group were randomly assigned to five primary groups. (1) For 7 weeks, the control group was given standard chow and 0.1 ml of saline intraperitoneally. The second group (CPZ) given cuprizone-mixed chow for 5 weeks and 0.1 ml of normal saline intraperitoneally. The third group received 0.1 ml of ZRO-based blank formulation administered intraperitoneally along with CPZ-mixed chow daily for 5 weeks. The fourth group was given CPZ-mixed chow and a ZRO-based formulation of curcumin (0.1 ml) intraperitoneally for 5 weeks. The fifth group was given CPZ-mixed chow and a ZRO-based nanoformulation of curcumin with piperine for 5 weeks. Cuprizone feeding was discontinued in all groups at the conclusion of the week 5 (cuprizone toxicity stage), but treatment with various nanoformulations persisted until the end of week 7. Group names and designations: (1) Control group, CNT; (2) Cuprizone-containing CPZ group; (3) Group given Cuprizone and blank formulation of ZRO, BFZ group; (4) Cuprizone-containing nanoformulation of curcumin, CFZ group; and (5) PFZ group (Cuprizone-containing curcumin and piperine formulation).

### 2.4 Y-maze spontaneous alternation test

The Y-maze spontaneous alternation task assesses short-term spatial working memory in mice. In this test, mice explore a Y-shaped apparatus (10 cm width and 40 cm height), composed of three identical arms, and separated equally by 120° angle for 8 min. Each mouse was placed at one arm-end facing the wall of the arm and allowed to explore the maze freely for 8 min. The percentage of spontaneous alternation is calculated based on the number of times a mouse visits all three arms consecutively (24). Arms entries and alternations between the arms were recorded to calculate the percentage of spontaneous alteration following the equation: % alternation = [Number of alternations/total arm visit −2] × 100.

### 2.5 Novel arm discrimination task

The Novel Arm Discrimination Task (NADT) evaluates spatial recognition memory using the Y-maze. It consists of two trials: acquisition and retention. During acquisition, one arm is blocked while mice explore the other two for 5 min. After 30 min, all arms are opened for a 5-min retention trial. Performance is measured by time spent and entries into the novel arm. The floor and the wall of the maze were wiped with 10% alcohol after each mouse to avoid odor cues. Time in seconds spent in the novel arm and total novel arm entries were calculated as an estimate for spatial recognition memory and mentioned in terms of percentage (27).

### 2.6 Novel object recognition test

To assess the impact of CPZ and other treatments on short-term recognition memory, the Novel Object Recognition Test (NORT) was conducted as previously described (24). The NORT consists of two phases: familiarization and testing. Prior to the familiarization phase, mice were habituated to an empty arena (45 cm × 45 cm square box) for 3 min. The following day, during the familiarization phase, mice explored two identical objects in the arena for 3 min. The test phase occurred 10 min later, with one familiar object replaced by a novel object of different shape and color. Mice allowed to explore the objects in a quiet environment for 3 min (28). Objects were chosen to be cleanable and heavy, and the arena and objects were cleaned with 10% ethanol between trials to eliminate odor cues. Sniffing frequency (%) was calculated for each object to ensure equal exploration opportunities. Memory was assessed using Novelty Preference (%), defined as the time spent exploring the novel object relative to total exploration time. An EthoVision tracking system (XT8A, Noldus Information Technology) recorded all parameters. Novelty Preference values range from 0% (no novel object exploration) to 100% (exclusive novel object exploration), with 50% indicating equal exploration of both objects.

### 2.7 Sample collection

By the conclusion of the fifth week, a random selection of 7 mice from each group were used to induce isoflurane anesthesia and decapitate them in order to end their lives. The whole brain of each mouse was then obtained by dissecting it. To get certain brain areas, including the hippocampus, frontal cortex, cerebral cortex, brain stem, and cerebellum, the obtained brains were further dissected. These brain slices were kept for future research at −80 °C after being immersed in RNA-later solution. At the conclusion of the study (week 7), the remaining mice (8 mice in each group) underwent the same process. Although samples were collected from multiple brain regions, this manuscript reports results from the hippocampus due to its central role in learning, memory, and neuroinflammatory processes.

### 2.8 Biochemical analysis

Tissues were homogenized in RIPA lysis buffer (R-0278; Sigma) containing PMSF at the prescribed doses, HaltTM phosphatase inhibitor (Thermo-Fisher Scientific), and protease inhibitor cocktail (cOmpleteTM, Roche) for use in various biochemical assays. After centrifuging the homogenate at 15000 × *g* for 20 min, the supernatant was separated and stored for further biochemical examination. With the use of a colorimetric assay kit obtained from SolarBio (Beijing, China), the SOD and CAT levels were determined. ELISA kits (colorimetric) from Sunlong Biotech Co., Ltd., (Hangzhou, China) were used to measure the levels of GSH, GPX, GFAP, MCP-1, MIP-1, MBP and CCL-5 in accordance with the manufacturer’s instructions.

### 2.9 Histological examinations

Paraffin-embedded tissue sections were cut at a thickness of 4–5 μm using a microtome and mounted on glass slides. The sections were subjected to Hematoxylin and Eosin (H&E) staining using standard histological procedures. Briefly, tissue sections were first deparaffinized in xylene for 10 min (2 changes, 5 min each) and then rehydrated through a graded ethanol series (100%, 95%, and 70%; 2 min each) to distilled water. Slides were immersed in hematoxylin solution for 5–10 min, followed by rinsing in running tap water for 2–5 min. Differentiation was performed using acid alcohol (1% HCl in 70% ethanol) for 5–10 s to remove excess stain, after which sections were rinsed in distilled water. To enhance nuclear staining, the sections were treated with a bluing agent (0.2% ammonia water) for 1–2 min and subsequently rinsed in tap water. Eosin staining was performed by immersing the slides in eosin Y solution for 30 s–2 min, depending on the desired staining intensity, followed by brief rinsing in distilled water. Dehydration of stained sections was achieved by passing the slides through ascending concentrations of ethanol (70%, 95%, and 100%; 1 min each), followed by clearing in xylene (2 changes, 3 min each). Finally, sections were mounted using a permanent mounting medium and covered with glass coverslips. Sections were examined under electrical microscope and photos were taken under a digital pathology Scanner. Hematoxylin stained the nuclei blue to dark purple, while eosin imparted varying shades of pink to the cytoplasm and extracellular matrix.

### 2.10 Statistical analysis

The changes and comparisons between different parameters at the end of toxicity stage (week 5) and healing stage (week 7) were statistically analyzed. Since the data were normally distributed, parametric tests [one-way ANOVA followed by Tukey’s *post-hoc* test also known as Tukey’s Honestly Significant Difference (HSD) test] were applied. This test was selected because all group comparisons were of equal interest and the design included balanced group sizes (*n* = 15 per group until week 5, and *n* = 8 per group until week 7). Tukey’s HSD provides robust control of Type I error across multiple comparisons while retaining greater statistical power compared to more conservative corrections such as Bonferroni, which may increase the risk of Type II errors in studies with modest sample sizes. This approach has been widely applied in preclinical animal research to ensure both rigor and sensitivity in detecting meaningful effects. Alterations in the results were considered significant when the *p*-value was ≤ 0.05. Most of the statistical analyses were performed using Microsoft Excel and Social Science Statistics freely available at https://www.socscistatistics.com/.

## 3 Results

### 3.1 Behavioral studies

#### 3.1.1 Spontaneous alteration test

**FIGURE 1:**
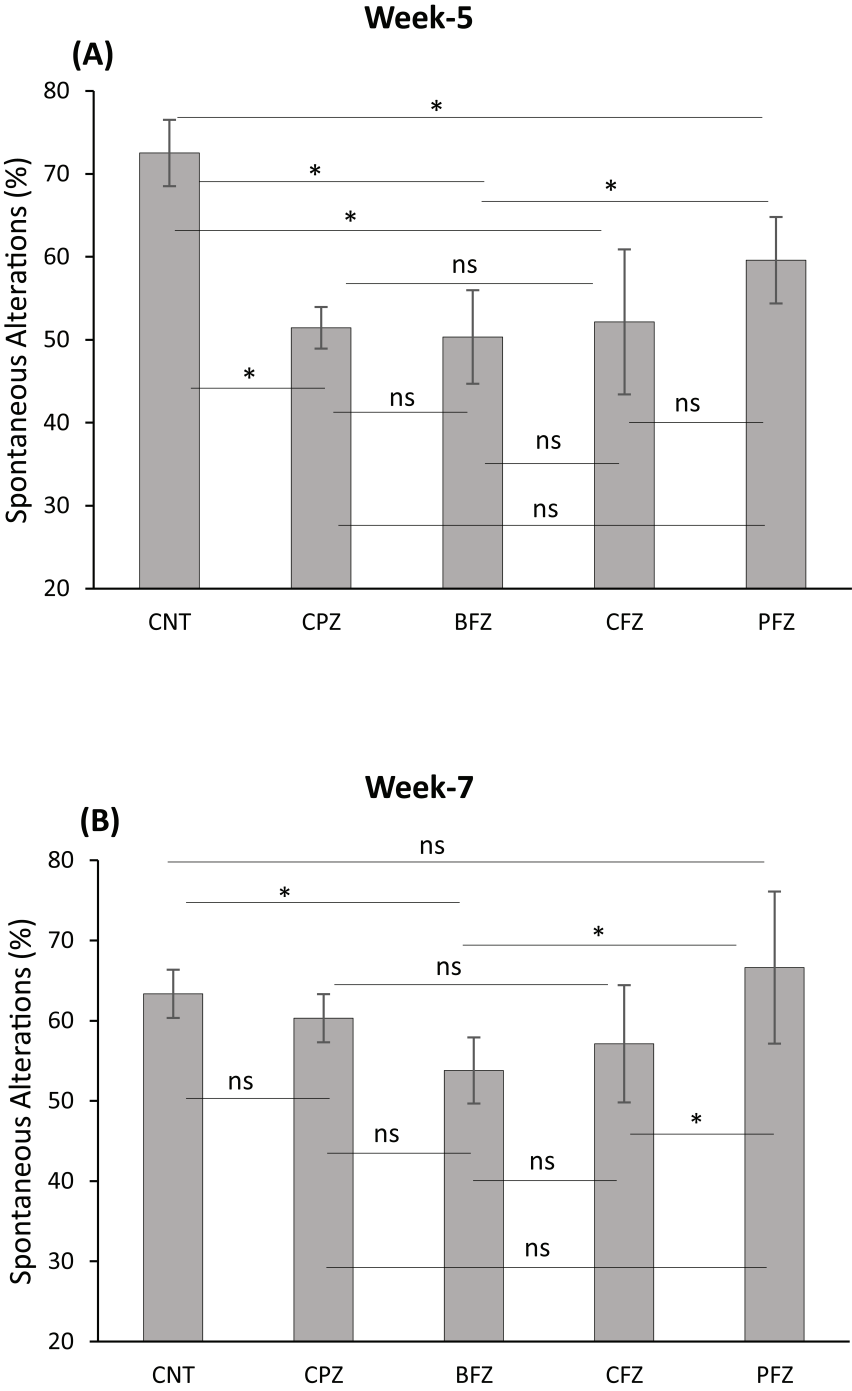
Y-maze spontaneous alteration task. **(A)** At the end of week 5 (demyelination); **(B)** at the end of week 7 (remyelination). Data are presented as mean ± SEM. One-way ANOVA with Tukey’s multiple comparison test was done to compare the variance between groups. *P ≤ 0.05; ns, non-significant.

Cuprizone feeding for 5 weeks resulted in a significant decrease (*p* ≤ 0.01) in short-term spatial working memory across all groups compared to the control group (Figure 1). There was no significant difference in percent spontaneous alterations between the treatment groups (BFZ, CFZ, and PFZ) and the CPZ group. By the end of the remyelination phase (week 7), the percent spontaneous alterations in the PFZ group were restored to levels similar to those of both the control and CPZ (now undergoing spontaneous remyelination) groups. Notably, the curcumin with piperine nanoformulation (PFZ) showed a significantly greater increase in percent spontaneous alterations compared to both the BFZ and CFZ groups (*p* ≤ 0.01) at week 7 (Figure 1).

#### 3.1.2 Novel arm discrimination task

**FIGURE 2:**
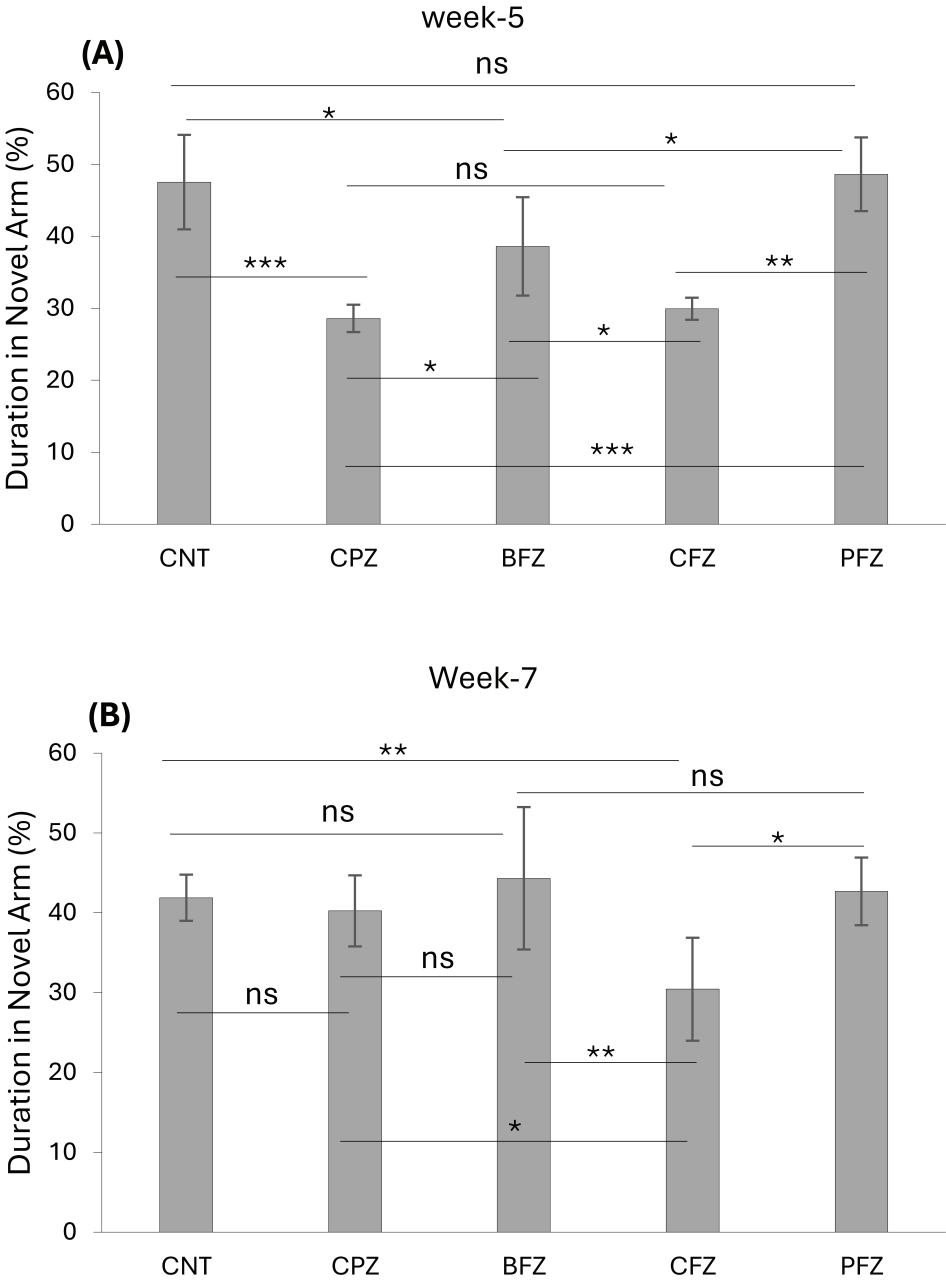
Novel arm discrimination task at panel **(A)** week 5 and **(B)** week 7. Data are presented as mean ± SEM. One-way ANOVA with Tukey’s multiple comparison test was done to compare the variance between groups. *P ≤ 0.05; **p ≤ 0.01; ***p ≤ 0.001; ns, non-significant.

**FIGURE 3:**
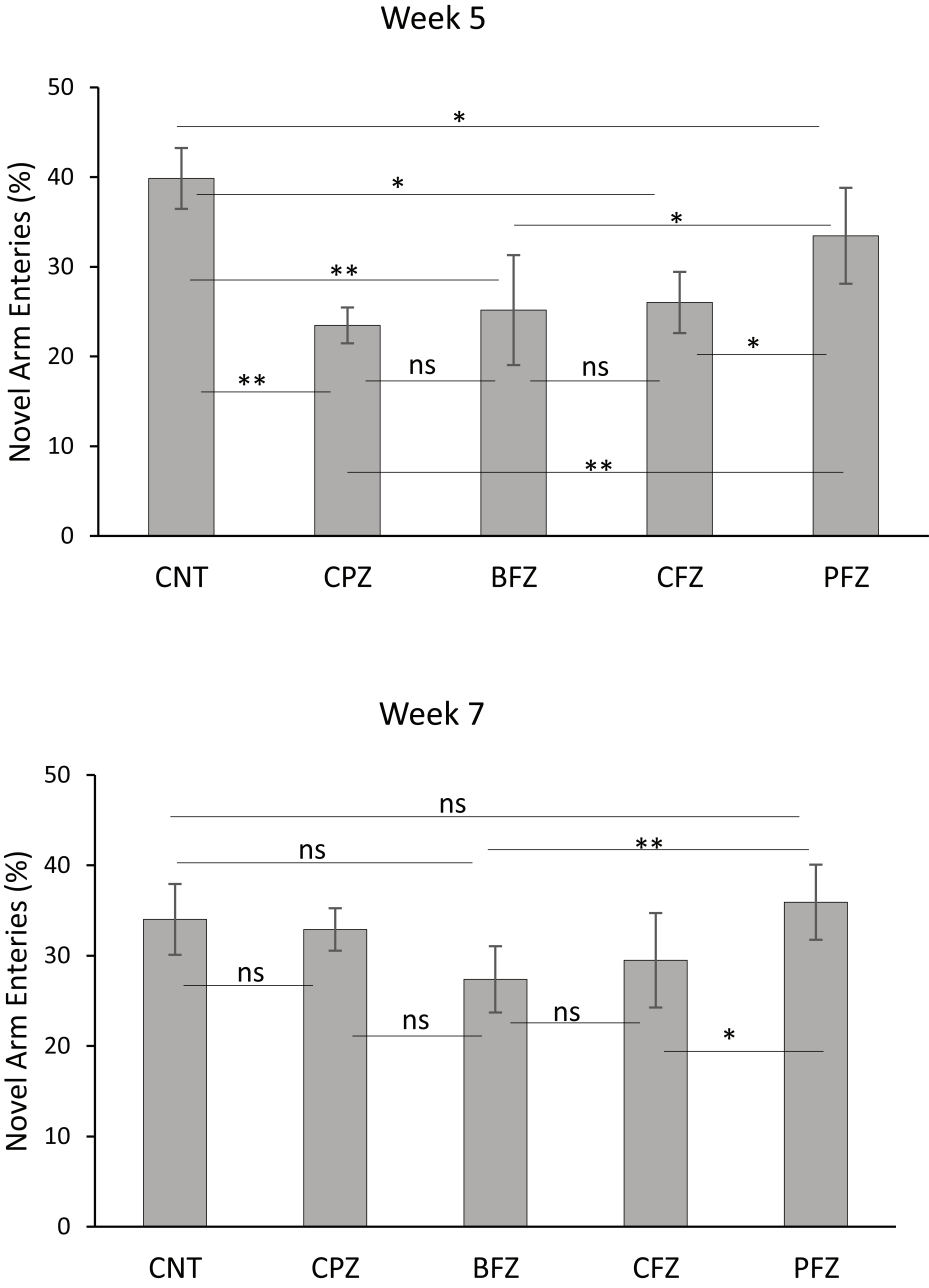
Percent entries into novel arm at week 5 and week 7. Data are presented as mean ± SEM. One-way ANOVA with Tukey’s multiple comparison test was done to compare the variance between groups. *P ≤ 0.05; **p ≤ 0.01; ns, non-significant.

At the end of the demyelination phase, mice in the CPZ group were found to spend significantly less time in the novel arm compared to the control group (*p* ≤ 0.001). Similarly, mice in the BFZ and CFZ treatment groups spent less time in the novel arm compared to the control group. In contrast, mice in the PFZ group spent a duration in the novel arm comparable to that of the control group. Additionally, mice in the BFZ and PFZ treatment groups spent significantly more time in the novel arm than those in the CPZ group (Figure 2). After 5 weeks of cuprizone feeding, cuprizone was removed from the diet in all groups, but treatment with the different formulations continued until the end of week 7. By the conclusion of week 7, there was no significant change in the duration spent in the novel arm by mice in the CNT and CPZ groups, or between the CNT and BFZ groups. Notably, mice in the curcumin treatment group (CFZ) spent significantly less time in the novel arm compared to the CPZ and PFZ groups (*p* ≤ 0.05) as well as the CNT and BFZ groups (*p* ≤ 0.01). Regarding the percentage of entries into the novel arm at week 5, mice in the CPZ group made significantly fewer entries (*p* ≤ 0.01) compared to the CNT group. Similarly, the treatment groups (BFZ, CFZ, and PFZ) also showed significantly fewer entries into the novel arm compared to the control group (Figure 3). Following two additional weeks without cuprizone, the pattern of novel arm entries was restored in the treatment groups, with values more similar to the control group (Figure 3).

#### 3.1.3 Novel object recognition test

**FIGURE 4:**
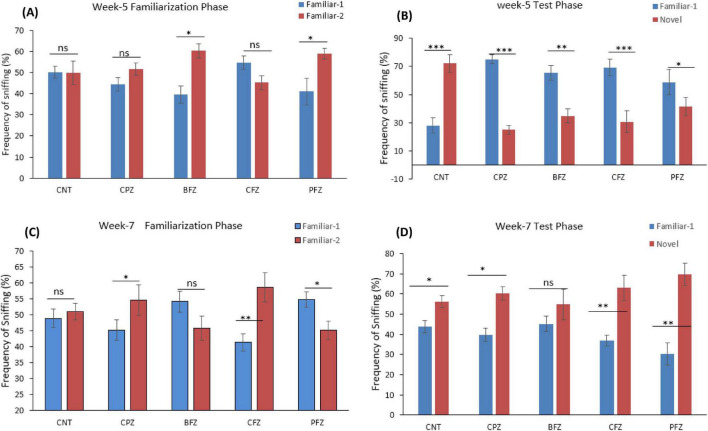
Frequency of sniffing (%) determined through novel object recognition test **(A)** familiarization phase at week 5; **(B)** test phase at week 5; **(C)** familiarization phase at week 7; **(D)** test phase at week 7. One-way ANOVA with Tukey’s multiple comparison test was done to compare the variance between groups. *P ≤ 0.05; **p ≤ 0.01; ***p ≤ 0.001; ns, non-significant.

**FIGURE 5:**
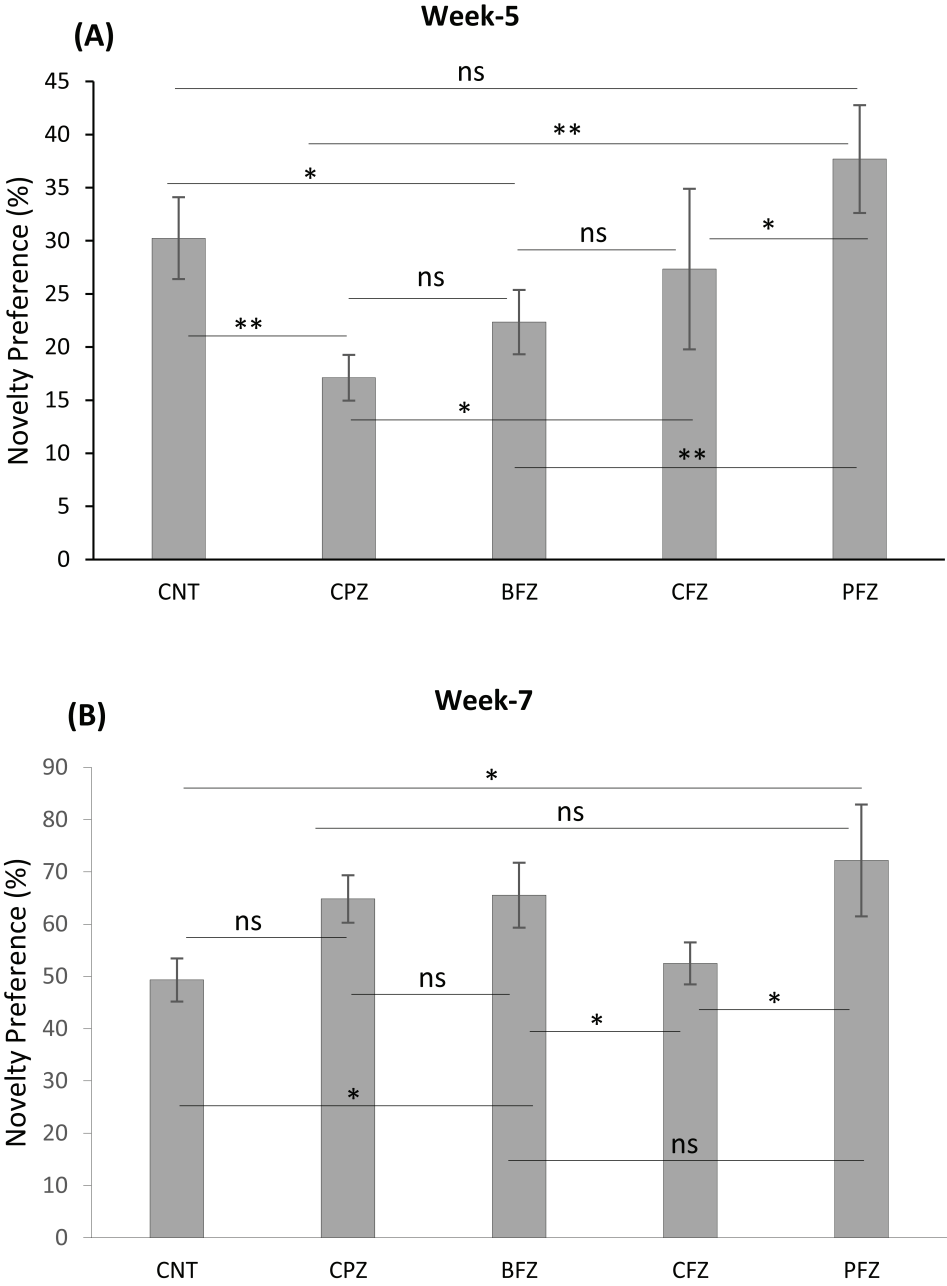
The analysis of novelty preference given as percent **(A)** at week 5 and **(B)** at week 7. Data are presented as mean ± SEM. One-way ANOVA with Tukey’s multiple comparison test was done to compare the variance between groups. *P ≤ 0.05; **p ≤ 0.01; ns, non-significant.

Apart from the BFZ and PFZ groups, no significant differences were observed in the frequency of sniffing familiar objects 1 and 2 during the familiarization phase at the end of the demyelination stage. However, during the test phase, significant differences in sniffing frequencies were observed across all groups. In the control group, the frequency of sniffing toward the novel object was significantly higher compared to the frequency for the familiar object (*p* ≤ 0.001). In contrast, all treatment groups showed significantly reduced sniffing frequencies toward the novel object when compared to the familiar object (Figure 4). The Novel Object Recognition Test (NORT) was repeated at the end of the remyelination stage (week 7). No significant difference in sniffing frequencies was observed between the familiar objects in the control and BFZ groups. However, in the CPZ and CFZ groups, a significant increase in sniffing frequency was observed toward familiar object 2, while the PFZ group showed a significant decrease in sniffing frequency for the familiar object (Figure 4). During the test phase, these results suggest that the withdrawal of cuprizone, combined with continued treatment with curcumin alone (CFZ) and curcumin with piperine (PFZ), significantly enhanced short-term recognition memory in mice. The analysis of novelty preference revealed that mice in the CPZ group exhibited significantly reduced preference for the novel object compared to the control group (*p* ≤ 0.01) at the end of week 5. Mice in the treatment groups demonstrated significantly better preference for the novel object compared to those in the CPZ group (Figure 5). By the conclusion of the experiment (week 7), mice in all treatment groups showed a significantly increased preference for the novel object compared to the control group (Figure 5).

### 3.2 Oxidative stress and antioxidant enzyme activity

#### 3.2.1 Effects of CPZ and nanoformulations on CAT level

The catalase levels across different experimental groups at the end of Week 5 and Week 7 are detailed in Table 1. After 5 weeks of cuprizone administration, the CPZ group exhibited a significant decline in catalase levels compared to the control, with a reduction of approximately 37.3%. This decline suggests that cuprizone induces oxidative stress, likely due to its known effects on glial activation, mitochondrial dysfunction, and oxidative damage, which contribute to the depletion of key antioxidant enzymes like catalase. Among the treatment groups, the BFZ group exhibited a lower catalase level (9.87 ± 0.84 U/mg) than CPZ but remained higher than CFZ and PFZ. The CFZ group demonstrated a substantial decrease in catalase levels, with a 71.3% reduction compared to CPZ and an 82% decrease relative to the control. This dramatic decline suggests severe oxidative stress despite curcumin administration, potentially due to inefficient delivery or increased metabolic demand. A similar trend was observed in the PFZ group, indicating that the addition of piperine did not enhance curcumin’s antioxidant effects as anticipated. Following the discontinuation of cuprizone after Week 5, treatment was continued for two additional weeks. By Week 7, catalase levels in the control group naturally declined by 50.2% from Week 5, possibly due to age-related oxidative stress. In the CPZ group, catalase remained significantly lower than the control, suggesting an absence of spontaneous recovery. Interestingly, the BFZ group exhibited higher catalase levels (11.28 ± 0.79 U/mg) than both CPZ and control groups. This suggests that the blank formulation may confer some protective effects against oxidative damage or facilitate compensatory upregulation of catalase activity. Notably, the BFZ group showed a 386% increase in catalase levels compared to CPZ, indicating a potential antioxidant role, though the precise mechanism remains unclear. The CFZ group (8.36 ± 0.51 U/mg protein) demonstrated a significant improvement in catalase levels, recording a 260% increase compared to CPZ. Meanwhile, the PFZ group (5.23 ± 0.27 U/mg protein) showed moderate recovery, with catalase levels surpassing CPZ but remaining lower than CFZ and BFZ. Although piperine is known to enhance curcumin’s bioavailability, its combination with curcumin in the PFZ group did not restore catalase levels as effectively as expected. However, both CFZ and BFZ displayed significant recovery by Week 7, with catalase levels increasing by 152.6% and 14.3%, respectively, compared to Week 5. These findings suggest that the formulations, particularly curcumin (CFZ), effectively reinstated antioxidant defenses over time following the withdrawal of cuprizone. Similarly, PFZ-treated animals exhibited a 39.5% increase in catalase levels compared to Week 5, though levels remained lower than CFZ and BFZ.

**TABLE 1:** Levels of antioxidants CAT, SOD, GSH and GPX in hippocampus at the end of week 5 and week 7.

Group	CAT (U/mg protein)				
	Week 5	*P*-value	Week 7	*P*-value	% change
CNT	18.41 ± 0.55	**** vs. CFZ	9.16 ± 0.42	** vs. CPZ	50.24↓
CPZ	11.55 ± 0.21	** vs. CNT, BFZ	2.32 ± 0.08	*** vs. BFZ	79.91↓
BFZ	9.87 ± 0.84	** vs. CFZ	11.28 ± 0.79	**** vs. PFZ * vs. CNT, CFZ	14.29↑
CFZ	3.31 ± 0.26	** vs. CPZ	8.36 ± 0.51	** vs. PFZ * vs. CPZ	152.56↑
PFZ	3.75 ± 0.34	**** vs. CNT	5.23 ± 0.27	** vs. CNT	39.46↑
	**SOD (U/mg protein)**				
	**Week 5**	***P*-value**	**Week 7**	***P*-value**	**% change**
CNT	16.38 ± 1.16	** vs. CPZ	29.26 ± 4.31	* vs. CPZ	78.63↑
CPZ	9.98 ± 0.75	* vs. CFZ	35.20 ± 2.99	** vs. PFZ	252.71↑
BFZ	16.54 ± 1.08	** vs. CPZ	24.60 ± 1.67	* vs. CNT	48.73↑
CFZ	14.29 ± 1.62	* vs. CNT	27.63 ± 2.03	* vs. CPZ	93.35↑
PFZ	14.67 ± 0.76	* vs. CNT	24.37 ± 1.84	* vs. CNT	66.12↑
	**GSH (ng/mg protein)**				
	**Week 5**	***P*-value**	**Week 7**	***P*-value**	**% change**
CNT	82.27 ± 4.05	** vs. PFZ; * vs. CPZ; NS vs. BFZ	42.96 ± 3.74	** vs. BFZ NS vs. PFZ	47.78↓
CPZ	53.04 ± 3.13	**** vs. PFZ	57.63 ± 9.87	* vs. CNT, CFZ	8.65↑
BFZ	85.25 ± 4.65	*** vs. PFZ * vs. CPZ	76.99 ± 5.21	** vs. PFZ	9.69↓
CFZ	91.14 ± 6.13	* vs. CPZ; NS vs. BFZ	80.22 ± 5.16	NS vs. BFZ	11.98↓
PFZ	132.88 ± 9.74	*** vs. CFZ	54.56 ± 4.17	** vs. CFZ	58.94↓
	**GPX (ng/mg protein)**				
	**Week 5**	***P*-value**	**Week 7**	***P*-value**	**% change**
CNT	1.37 ± 0.06	*** vs. BFZ * vs. CPZ	1.29 ± 0.02	NS vs. CPZ	5.84↓
CPZ	0.74 ± 0.03	**** vs. BFZ	1.52 ± 0.07	* vs. CNT, BFZ	105.41↑
BFZ	2.06 ± 0.26	** vs. CFZ NS vs. PFZ	2.01 ± 0.08	** vs. PFZ	2.43↓
CFZ	1.95 ± 0.19	** vs. CNT, CPZ	1.65 ± 0.11	** vs. PFZ	15.38↓
PFZ	5.78 ± 0.31	** vs. CNT	5.39 ± 0.10	** vs. CNT	6.75↓

One-way ANOVA with Tukey’s multiple comparison test was done to compare the variance between groups.

*P ≤ 0.05, **p ≤ 0.01, ***p ≤ 0.001, ****p ≤ 0.0001; ns, non-significant. ↑ and ↓ represent increase and decrease in amount of protein respectively.

#### 3.2.2 Effects of CPZ and nanoformulations on SOD level

The SOD levels recorded in Week 5 and Week 7 are summarized in Table 1. At the end of Week 5, the control group exhibited an SOD level of 16.38 ± 1.16 U/mg, reflecting normal antioxidant activity. In contrast, the CPZ group showed a substantial decrease in SOD levels, measuring 9.98 ± 0.75 U/mg, which represents a 39% reduction compared to the control. This decline highlights the severe oxidative stress induced by cuprizone toxicity. Among the treatment groups, BFZ (16.54 ± 1.08 U/mg) maintained SOD levels similar to the control, suggesting that the blank formulation may have conferred some protective effects against oxidative damage. On the other hand, CFZ (14.29 ± 1.62 U/mg) and PFZ (14.67 ± 0.76 U/mg) showed moderate reductions in SOD activity compared to the control, indicating that curcumin and piperine had not yet exerted a substantial antioxidant effect. By Week 7, all groups exhibited a significant rise in SOD levels, with the most striking increase observed in the CPZ group. SOD levels in CPZ surged by 252.7%, rising from 9.98 ± 0.75 to 35.20 ± 2.99 U/mg. Among the treatment groups, CFZ exhibited the most pronounced improvement, with a 93.4% increase in SOD levels by Week 7, suggesting that curcumin played a significant role in enhancing antioxidant defenses. The PFZ group also showed notable recovery (66.1% increase), though the effect was less pronounced than in CFZ, indicating that while piperine improves curcumin bioavailability, its impact on SOD restoration may be limited. BFZ displayed the least increase in SOD activity (48.7%), yet its levels remained higher than CPZ at Week 5, reinforcing the idea that the blank formulation may have some inherent protective properties.

#### 3.2.3 Effects of CPZ and nanoformulations on GSH level

The present study assessed GSH levels at Weeks 5 and 7 across different experimental groups to evaluate the impact of cuprizone (CPZ) exposure and potential neuroprotective interventions. At Week 5, the control group exhibited a GSH concentration of 82.27 ± 4.05 ng/mg protein, reflecting a normative antioxidant state. Conversely, the CPZ-exposed group demonstrated a marked reduction in GSH levels (53.04 ± 3.13 ng/mg protein), indicative of significant oxidative stress induced by cuprizone exposure. This depletion aligns with prior research highlighting GSH reduction as a key characteristic of cuprizone-mediated neurotoxicity (29). Among the treatment groups, the PFZ cohort exhibited the highest GSH concentration (132.88 ± 9.74 ng/mg protein), significantly surpassing the control group, suggesting that piperine-enhanced curcumin may facilitate GSH synthesis. The CFZ (91.14 ± 6.13 ng/mg protein) and BFZ (85.25 ± 4.65 ng/mg protein) groups also demonstrated elevated GSH levels relative to both the CPZ and control groups, further indicating potential neuroprotective effects. By the conclusion of Week 7, the control group exhibited a substantial reduction in GSH levels (a 47.8% decline), likely reflecting an age-related decrease in antioxidant capacity. Notably, the CPZ group showed a slight increase in GSH levels (57.63 ± 9.87 ng/mg protein), corresponding to an 8.6% recovery, suggesting a partial endogenous response following the cessation of cuprizone exposure. However, this increase was not statistically significant, implying that oxidative stress persisted despite treatment discontinuation. The BFZ and CFZ groups experienced moderate reductions in GSH (9.7% and 12%, respectively) but maintained levels exceeding those of the control group, indicative of sustained antioxidant benefits. The PFZ group, however, exhibited the most pronounced decline in GSH levels (58.9%), decreasing from 132.88 ± 9.74 to 54.56 ± 4.17 ng/mg protein, which fell below the control group level. This sharp decline suggests that the initially elevated GSH concentrations observed in PFZ-treated subjects may not be maintained over time, potentially due to regulatory feedback mechanisms or metabolic exhaustion of GSH synthesis pathways.

#### 3.2.4 Effects of CPZ and nanoformulations on GPX level

The GPX levels for different experimental groups at two time points are summarized in the Table 1. At Week 5, the control group exhibited a GPX level of 1.37 ± 0.06 ng/mg protein, representing normal antioxidant enzyme activity. In contrast, the CPZ group showed significantly lower GPX level (0.74 ± 0.03 ng/mg protein), indicating severe oxidative stress. PFZ exhibited the highest GPX level (5.78 ± 0.31 ng/mg protein), which was nearly four times higher than the control group, suggesting a strong enhancement of antioxidant defense, possibly due to piperine-enhanced curcumin’s ability to upregulate GPX expression. BFZ (2.06 ± 0.26 ng/mg protein) and CFZ (1.95 ± 0.19 ng/mg protein) also had elevated GPX levels compared to the control, indicating a protective effect against cuprizone-induced oxidative stress. At the end of Week 7, the control group showed a slight reduction in GPX activity (5.8% decline), which is consistent with age-related declines in antioxidant enzyme activity. However, a notable trend was observed in the other groups. The CPZ group showed a significant increase in GPX activity (from 0.74 ± 0.03 to 1.52 ± 0.07 ng/mg protein, a 105.4% increase), suggesting some degree of spontaneous recovery following CPZ withdrawal. However, despite this increase, the CPZ group’s GPX level remained lower than most treatment groups, indicating persistent oxidative stress. BFZ, CFZ and PFZ showed only mild reductions in GPX maintaining relatively high levels of antioxidant defense.

### 3.3 Neuroinflammatory markers

#### 3.3.1 Effects of CPZ and nanoformulations on GFAP level

Glial fibrillary acidic protein levels were measured in different experimental groups at the conclusion of Week 5 and Week 7. The results are presented in Table 2. The data indicate that all experimental groups (CPZ, BFZ, CFZ, and PFZ) exhibited increased GFAP expression compared to the Control group, suggesting heightened astrocytic activation. The Control group had the lowest GFAP levels (32.31 ± 3.42 pg/mg protein), representing a normal physiological state without astrocytic activation. All treated groups (CPZ, BFZ, CFZ, and PFZ) showed higher GFAP levels, indicating varying degrees of neuroinflammatory responses. The administration of cuprizone for 5 weeks resulted in significant increase in the level of GFAP in CPZ group (56.55 ± 1.9 pg/mg protein). The nanoformulations of curcumin alone (CFZ) curcumin with piperine (PFZ) and blank formulation appears to fail protective effect against cuprizone toxicity as GFAP levels in these groups observed to be significantly higher than the CPZ group. At the end of week 5, cuprizone was withdrawn from the rodent diet but the treatment with nanoformulations continued for further 2 weeks. At the end of week 7, notable differences were observed in GFAP levels across groups. The Control group had the lowest GFAP levels at both time points (week 5 and week 7), with a slight reduction from Week 5 (32.31 ± 3.42) to Week 7 (29.17 ± 3.94), indicating a stable baseline of astrocytic activity. The CPZ group showed a decline in GFAP levels from Week 5 (56.55 ± 1.9) to Week 7 (44.28 ± 4.23), suggesting a potential spontaneous reduction in astrocytic activation since cuprizone was not given after week 5. The PFZ group showed a significant reduction in GFAP levels, decreasing from 51.33 ± 2.15 at Week 5 to 11.53 ± 1.14 at Week 7, suggesting the beneficial impact of the curcumin with piperine nanoformulation in reducing astrocytic activation. Similarly, the CFZ group experienced a decline from 48.34 ± 5.9 at Week 5 to 21.45 ± 2.68 at Week 7, indicating therapeutic effects of only curcumin formulations in reducing the neuroinflammatory response.

**TABLE 2:** Levels of inflammatory markers GFAP, MCP-1, MIP-1 and CCL-5 in hippocampus at the end of week 5 and week 7.

Group	GFAP (pg/mg protein)				
	Week 5	*P*-value	Week 7	*P*-value	% change
CNT	32.31 ± 3.42	**** vs. PFZ	29.17 ± 3.94	* vs. CFZ	9.72↓
CPZ	56.55 ± 1.9	*** vs. CNT	44.28 ± 4.23	**** vs. PFZ	21.69↓
BFZ	51.33 ± 2.15	*** vs. CNT	65.84 ± 3.25	**** vs. PFZ	28.26↑
CFZ	48.34 ± 5.9	** vs. CNT	21.45 ± 2.68	**** vs. BFZ	55.62↓
PFZ	59.28 ± 3.37	* vs. CFZ, BFZ	11.53 ± 1.14	** vs. CPZ	80.55↓
	**MCP-1 (pg/mg protein)**				
	**Week 5**	***P*-value**	**Week 7**	***P*-value**	**% change**
CNT	176.56 ± 13.42	*** vs. CPZ	311.26 ± 23.94	** vs. CPZ	76.29↑
CPZ	489.35 ± 33.37	*** vs. PFZ	627.94 ± 40.35	*** vs. BFZ, CFZ	28.32↑
BFZ	328.12 ± 22.15	* vs. CNT, CPZ	251.94 ± 31.14	* vs. CNT, PFZ	23.22↓
CFZ	327.40 ± 25.9	* vs. PFZ	213.77 ± 22.68	* vs. PFZ	34.71↓
PFZ	175.48 ± 10.17	** vs. BFZ	358.81 ± 54.32	** vs. CPZ	104.47↑
	**MIP-1 (pg/mg protein)**				
	**Week 5**	***P*-value**	**Week 7**	***P*-value**	**% change**
CNT	211.96 ± 25.34	*** vs. CPZ	140.11 ± 19.64	** vs. CPZ	33.89↓
CPZ	501.06 ± 39.41	**** vs. CFZ	274.32 ± 33.86	* vs. CFZ	45.25↓
BFZ	57.84 ± 6.38	**** vs. CPZ	136.71 ± 17.16	*** vs. CPZ	136.35↑
CFZ	57.02 ± 11.44	** vs. CNT	198.53 ± 26.14	* vs. BFZ	248.17↑
PFZ	66.38 ± 9.61	**** vs. CPZ	170.79 ± 21.35	* vs. PFZ	157.29↑
	**CCL-5 (pg/mg protein)**				
	**Week 5**	***P*-value**	**Week 7**	***P*-value**	**% change**
CNT	539.02 ± 63.57	** vs. CPZ	511.33 ± 45.36	** vs. CPZ	5.19↓
CPZ	935.04 ± 119.31	NS vs. BFZ	1065.62 ± 135.94	* vs. BFZ	13.96↑
BFZ	883.07 ± 89.65	** vs. CNT	718.69 ± 88.74	NS vs. PFZ	18.61↓
CFZ	591.05 ± 49.71	* vs. BFZ	726.78 ± 69.58	* vs. CPZ	22.96↑
PFZ	553.64 ± 51.23	NS vs. CFZ	604.58 ± 52.47	* vs. CPZ	9.20↑

One-way ANOVA with Tukey’s multiple comparison test was done to compare the variance between groups.

*P ≤ 0.05, **p ≤ 0.01, ***p ≤ 0.001, ****p ≤ 0.0001; ns, non-significant. ↑ and ↓ represent increase and decrease in amount of protein respectively.

#### 3.3.2 Effects of CPZ and nanoformulations on MCP-1 level

Monocyte chemoattractant protein-1 levels were measured at various time points across different experimental groups. The percentage changes between these time points are summarized in Table 2. After 5 weeks of cuprizone feeding, a significant increase in MCP-1 levels was observed in the CPZ group compared to the control group. Specifically, MCP-1 expression rose by 177.1%, indicating a robust inflammatory response linked to cuprizone-induced neurotoxicity. The blank formulation (BFZ) and curcumin-alone formulation (CFZ) both partially reduced cuprizone-induced inflammation. Although MCP-1 levels remained higher than the control group, they were significantly lower than in the CPZ group, suggesting a moderate neuroprotective effect. BFZ reduced MCP-1 levels by 32.95% compared to the CPZ group, while CFZ showed a 33.09% reduction, demonstrating their ability to mitigate neuroinflammatory responses. The piperine-curcumin formulation (PFZ) led to a significant decrease in MCP-1 levels, almost returning to control values. This suggests that piperine enhances curcumin’s neuroprotective effects potentially improving curcumin’s bioavailability and anti-inflammatory properties. By the conclusion of Week 7, MCP-1 levels in the CPZ group continued to rise from 489.35 ± 33.37 pg/mg protein to 627.94 ± 40.35 pg/mg protein, indicating a lack of spontaneous recovery without treatment. In contrast, the BFZ and CFZ groups showed a significant decrease in MCP-1 levels compared to the CPZ group, suggesting a faster recovery due to the nanoformulations. This indicates the potential of these formulations in promoting neurorepair and reducing inflammation. In the PFZ group, MCP-1 levels showed a slight, non-significant increase compared to the control group, further emphasizing its strong protective role in counteracting cuprizone-induced neuroinflammation.

#### 3.3.3 Effects of CPZ and nanoformulations on MIP-1 level

The CPZ group exhibited the highest MIP-1 levels (501.06 ± 39.41 pg/mg protein), significantly higher than the control group (211.96 ± 25.34 pg/mg protein) when measured at the end of week 5. This sharp increase indicates that cuprizone feeding triggers a strong inflammatory response, likely driven by microglial activation and astrocytic proliferation in the brain. In comparison, the treatment groups (BFZ, CFZ, and PFZ) had notably lower MIP-1 levels than the CPZ group (Table 2). MIP-1 levels in the BFZ and CFZ groups were nearly identical, suggesting both formulations effectively suppressed CPZ-induced inflammation. The PFZ group (66.38 ± 9.61 pg/mg protein) exhibited slightly higher MIP-1 levels than BFZ and CFZ, implying that piperine may not significantly enhance the anti-inflammatory effect of curcumin regarding MIP-1 levels. At the conclusion of the study (Week 7), MIP-1 levels had changed markedly across all groups. The CPZ group showed a significant decrease in MIP-1 levels (274.32 ± 33.86 pg/mg protein, a 45.25% reduction compared to Week 5), but the levels still remained much higher than those in the control group, indicating ongoing inflammation despite the cessation of cuprizone exposure.

#### 3.3.4 Effects of CPZ and nanoformulations on CCL-5 level

The CPZ group had the highest CCL-5 levels (935.04 ± 119.31 pg/mg protein), which were significantly higher compared to the control group (Table 2) at the end of Week 5. This considerable increase in CCL-5 indicates that cuprizone exposure triggers a strong neuroinflammatory response. The BFZ treatment group showed lower CCL-5 levels than the CPZ group, but still higher than the control group. In contrast, the CCL-5 levels in the CFZ and PFZ groups were similar to those in the control group, suggesting that these nanoformulations have anti-inflammatory effects. After cuprizone was removed from the rodent diet, significant changes in CCL-5 levels were observed across all groups by the end of Week 7.

D Demyelination and myelin preservation

#### 3.3.5 Effects of CPZ and nanoformulations on MBP level

To evaluate the effect of different treatment regimens on cuprizone-induced demyelination, hippocampal levels of Myelin Basic Protein (MBP) were assessed at Weeks 5 and 7 (Table 3). At Week 5, animals exposed to cuprizone (CPZ group) exhibited a marked reduction in MBP (21.10 ± 1.74 pg/mg protein) compared with the control group (CNT, 31.88 ± 2.92 pg/mg protein; *p* < 0.01), confirming substantial demyelination. Treatment with BFZ (27.48 ± 1.26 pg/mg protein) partially restored MBP and was significantly different from both CNT and CPZ (*p* < 0.05). The CFZ group (29.05 ± 2.42 pg/mg protein) also demonstrated a significant recovery relative to CPZ (*p* < 0.01), although levels remained below those of CNT. Notably, PFZ produced the strongest protective effect, as MBP levels were elevated to 37.65 ± 3.68 pg/mg protein, which was significantly higher than CNT (*p* < 0.05). By Week 7, MBP levels in the CPZ group remained significantly reduced (19.25 ± 1.22 pg/mg protein) and were markedly lower than PFZ (*p* < 0.0001). Similarly, the BFZ group (21.22 ± 1.86 pg/mg protein) showed persistently diminished MBP levels compared with CNT (*p* < 0.05). In contrast, CFZ (26.18 ± 2.48 pg/mg protein) maintained significantly higher MBP than CPZ (*p* < 0.05) and PFZ (*p* < 0.001), indicating moderate protection. The PFZ group showed the most pronounced effect, with MBP levels rising to 42.86 ± 3.42 pg/mg protein, significantly higher than CNT (*p* < 0.0001), suggesting robust enhancement of myelin preservation. When evaluating changes between Weeks 5 and 7, the CNT group showed a natural decline of 45.60% in MBP, while CPZ exhibited a further reduction of 8.76%, reflecting progressive demyelination. Both BFZ and CFZ demonstrated declines of 22.78% and 9.88%, respectively. Strikingly, PFZ was the only group to exhibit a positive change, with MBP levels increasing by 13.84% over time, highlighting its potent myelin-protective capacity.

**TABLE 3:** Levels of Myelin Basic Protein (MBP) in hippocampus at the end of week 5 and week 7.

Group	MBP (pg/mg protein)				
	Week 5	*P*-value	Week 7	*P*-value	% change
CNT	31.88 ± 2.92	** vs. CPZ	17.34 ± 0.98	** vs. CFZ	45.60↓
CPZ	21.10 ± 1.74	*** vs. PFZ	19.25 ± 1.22	**** vs. PFZ	8.76↓
BFZ	27.48 ± 1.26	* vs. CNT, CPZ	21.22 ± 1.86	* vs. CNT	22.78↓
CFZ	29.05 ± 2.42	** vs. CPZ, PFZ	26.18 ± 2.48	*** vs. PFZ; * vs. CPZ	9.88↓
PFZ	37.65 ± 3.68	* vs. CNT	42.86 ± 3.42	**** vs. CNT	13.84↑

One-way ANOVA with Tukey’s multiple comparison test was done to compare the variance between groups.

*p ≤ 0.05, **p ≤ 0.01, ***p ≤ 0.001, ****p ≤ 0.0001; ns, non-significant. ↑ and ↓ represent increase and decrease in amount of protein respectively.

### 3.4 Histological examination

#### 3.4.1 Hematoxylin and Eosin staining

**FIGURE 6:**
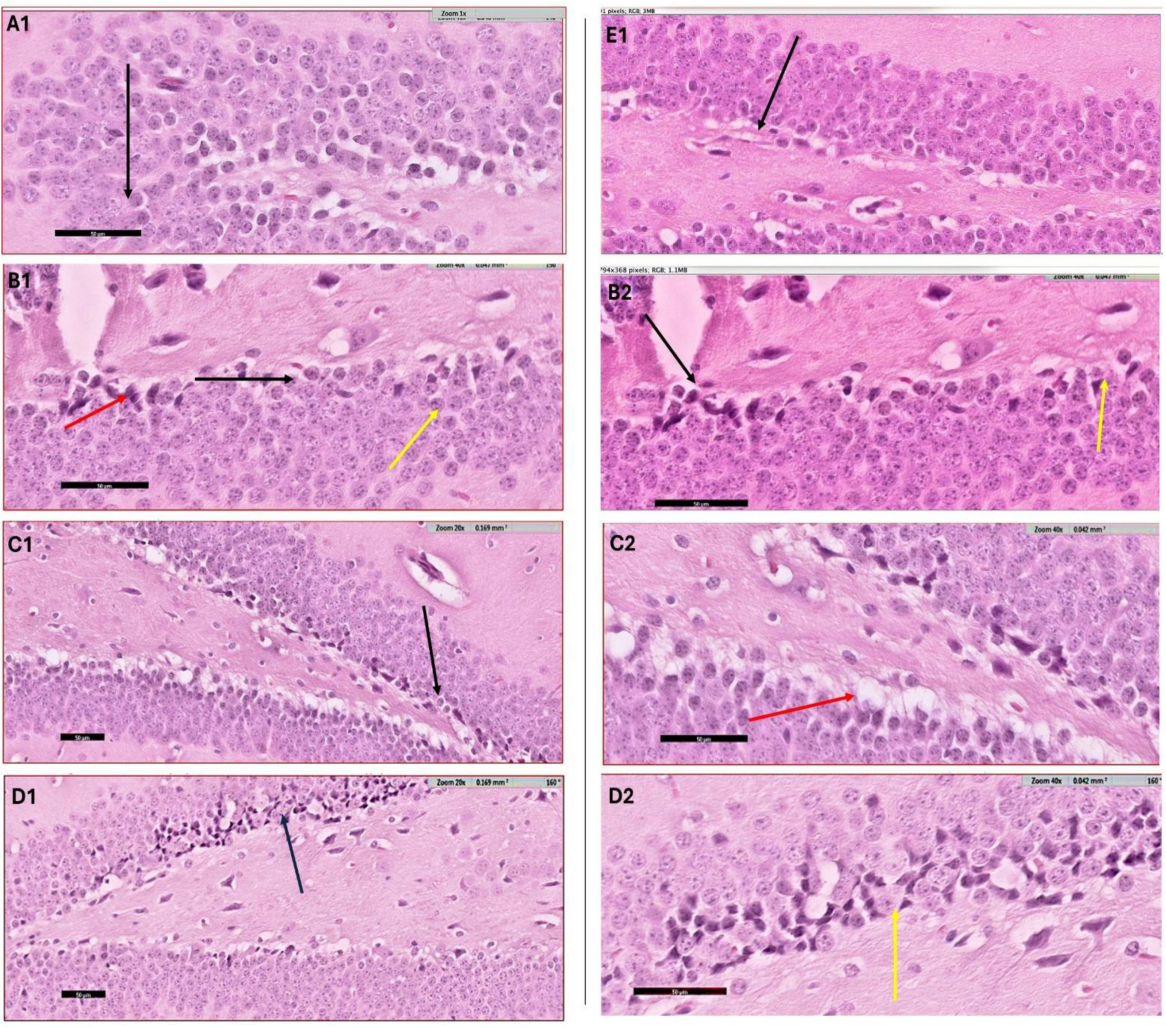
H&E staining results – week 5. Photomicrograph of dentate gyrus region of hippocampus at week 5: **(A1)** control mice showing the normal architecture of dentate region (black arrow) formed mainly of aggregation of granular cells. **(B1)** CPZ group mice showing dentate gyrus (black arrow) with marked loss of its normal architecture (loss of granular layer) and marked decrease in thickness with apparently packed cells, notice the shrunken cells (yellow arrow) as well as ruptured one (red arrow). Same image is captured at higher magnification as depicted in panel **(B2)**. **(C1)** Brain from BFZ group mice showing some vacuolated cells in the granular cell layer (black arrow) and same image is shown in higher magnification in panel **(C2)**. **(D1)** Brain section from CFZ group mice showing dentate gyrus disturbed areas of granular cell layer (black arrow) and yellow arrow in magnified image **(D2)**. **(E1)** PFZ mice showing the normal architecture of dentate region formed mainly of aggregation of granular cells.

**FIGURE 7:**
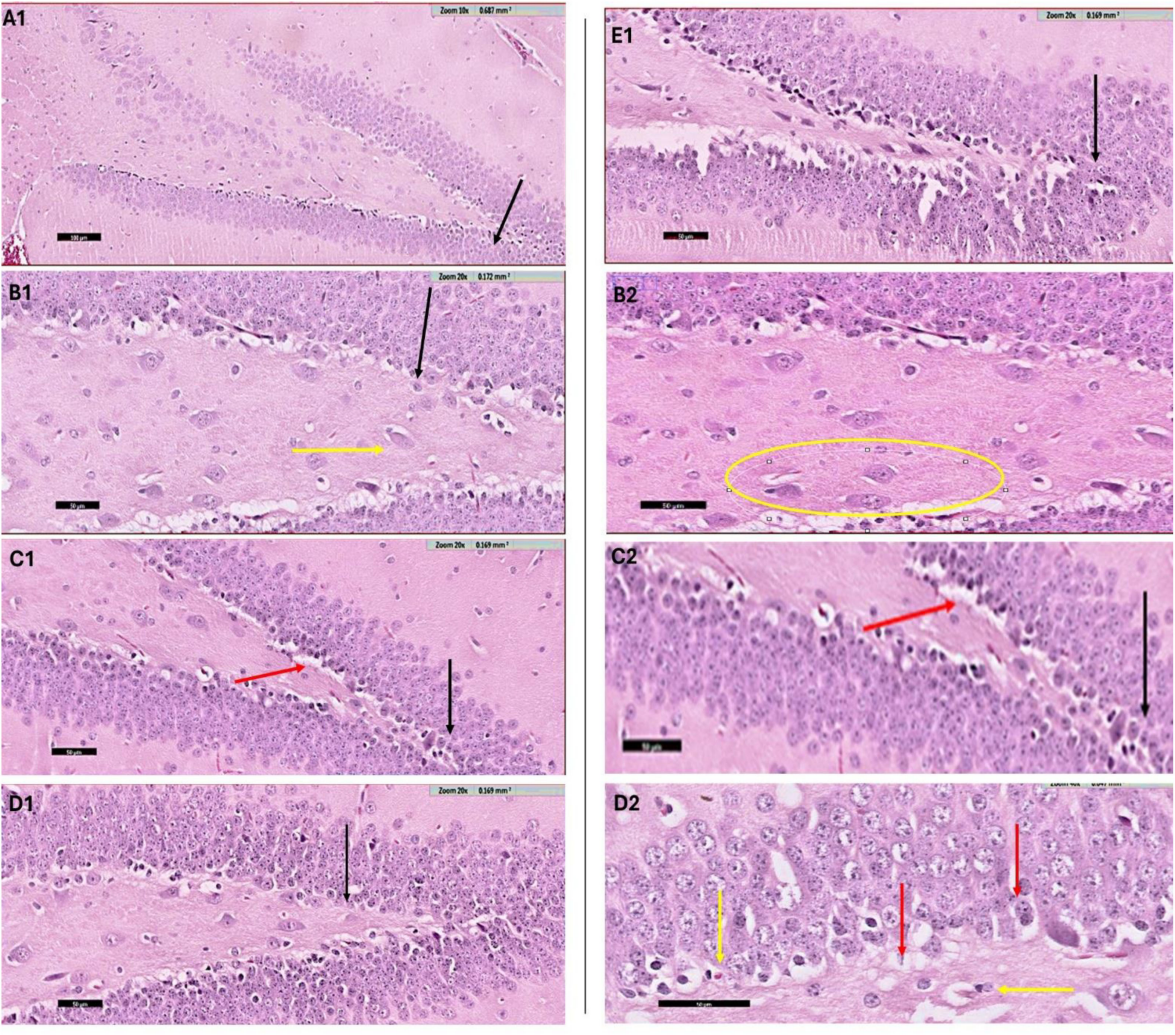
H&E staining results – week 7. Photomicrograph of dentate gyrus region of hippocampus (H & E staining) at week 7: **(A1)** control mice showing the normal architecture of dentate region (black arrow) formed mainly of aggregation of granular cells. **(B1)** CPZ treated mice showing dentate gyrus (black arrow) with mild loss of its normal architecture, notice the vacuolated cytoplasm of the basal granular cell layer (yellow arrow). A selected region of same image is shown magnified in panel **(B2)**. **(C1)** BFZ mice showing the normal architecture of dentate region (black arrow) formed mainly of aggregation of granular cells with mild loss of basal layer (red arrow), **(C2)** is magnified image of the area of interest. **(D1)** Section from CFZ group mice showing dentate gyrus (black arrow) with normal architecture. In its magnified image **(D2)** few disturbed areas of granular cell layer (red arrow) and pyknotic nuclei (yellow arrow) are shown. **(E1)** PFZ mice showing the normal architecture of dentate region (black arrow) formed mainly of aggregation of granular cells.

The histological evaluation of the dentate gyrus region of the hippocampus using hematoxylin and eosin (H&E) staining provided insight into the progression of CPZ-induced neurotoxicity and the potential neuroprotective effects of various nanoformulated treatments over time. At Week 5, CPZ-treated mice exhibited pronounced structural disruption in the dentate gyrus, including loss of the granular layer, increased tissue thickness, and evidence of degenerating cells such as shrunken and ruptured neurons (Figure 6). In contrast, mice treated with BFZ and CFZ showed partial preservation of structure, with limited vacuolization and disorganized areas of the granular layer, respectively. These mild histopathological features suggest a degree of neuroprotection, potentially due to anti-inflammatory or antioxidant properties of the formulations used. Notably, PFZ-treated mice displayed preserved histoarchitecture similar to the control group, indicating strong neuroprotective efficacy in maintaining cellular organization and preventing CPZ-induced damage (Figure 6). By Week 7, histological recovery or stabilization became more apparent across the treatment groups (Figure 7). The CPZ group showed mild residual damage, including vacuolated cytoplasm, but with reduced severity compared to Week 5. The BFZ and CFZ groups demonstrated near-normal architecture, with only minor disturbances such as pyknotic nuclei, suggesting an ongoing neuroprotective or reparative effect. The PFZ group continued to show well-preserved granular cell layers, reinforcing its consistent and potent neuroprotective profile (Figure 7). The temporal improvement observed in treated groups implies that the nanoformulated compounds may mitigate CPZ-induced neurotoxicity by preserving neuronal integrity and possibly enhancing repair mechanisms.

## 4 Discussion

### 4.1 Behavioral studies

Cuprizone disrupts cellular metabolism by chelating copper, an essential cofactor for numerous enzymatic processes, including antioxidant defense and myelin maintenance. This disruption leads to mitochondrial dysfunction and oxidative stress, ultimately causing the death of oligodendrocytes, the cells responsible for producing myelin (7). The primary consequence of cuprizone toxicity is the demyelination of specific brain regions, such as the corpus callosum and hippocampus. This process is accompanied by an inflammatory response involving microglia and astrocytes (30) and impairs the conduction of electrical signals between neurons (31). Microglia and macrophages accumulate in affected areas, contributing to further neuronal damage. Research into cuprizone-induced neurotoxicity emphasizes the importance of antioxidants and anti-inflammatory agents in protecting against neurodegeneration. In this study, we used cuprizone as a neurotoxicant for mice and investigated the prophylactic and therapeutic effects of piperine and curcumin nanoformulations prepared with *Zanthoxylum rhetsa* seed oil. Curcumin’s health benefits are largely attributed to its antioxidant and anti-inflammatory properties. However, curcumin alone has poor bioavailability, primarily due to limited absorption, rapid metabolism, and quick elimination. Components such as piperine have been shown to enhance curcumin’s bioavailability (13).

To assess the effects of cuprizone, as well as the blank and nanoformulations prepared with *Zanthoxylum rhetsa* seed oil, on spatial and recognition memory, we conducted several behavioral tests on mice such as the Y-maze, Novel Object Recognition Test (NORT), and Novel Arm Discrimination Test (NADT) was intentional, as these paradigms probe different but complementary domains of memory that are critically dependent on hippocampal integrity. The Y-maze task is widely employed to assess spatial working memory through spontaneous alternation behavior, which reflects the rodent’s innate drive to explore novel environments. Successful alternation requires intact hippocampal – prefrontal connectivity and synaptic plasticity, processes that are highly vulnerable to demyelination, oxidative stress, and neuroinflammation induced by cuprizone exposure. Thus, impaired Y-maze performance is a sensitive indicator of hippocampal dysfunction and reduced cognitive flexibility (32). The NORT evaluates recognition memory based on the natural preference of rodents for novel objects over familiar ones (33). Recognition memory depends on hippocampal–perirhinal cortical networks, and deficits in this task have been consistently reported in models of neurodegeneration, oxidative stress, and synaptic impairment (34). In the cuprizone model, the oxidative and inflammatory milieu disrupts neuronal signaling and hippocampal synaptic integrity, thereby leading to marked impairments in recognition-based learning that are readily detected using the NORT.

In addition, the NADT was included to provide a more refined measure of spatial recognition and memory consolidation, as it requires the animal to discriminate between previously explored and novel arms of the maze. This task engages hippocampal circuits responsible for encoding and consolidating spatial information, and impairments reflect deficits in long-term memory processing, which are exacerbated by demyelination and glial activation (32). The NADT therefore complements the Y-maze and NORT by offering insight into hippocampal-dependent long-term memory domains that may respond differently to therapeutic interventions. Taken together, the combination of these behavioral tasks provides a comprehensive assessment of hippocampal-dependent memory functions, capturing impairments in spatial working memory, recognition-based learning, and long-term memory consolidation. This multidimensional approach not only validates the cognitive consequences of cuprizone-induced neurotoxicity but also highlights the therapeutic potential of curcumin–piperine nanoparticles in restoring hippocampal integrity and improving behavioral outcomes.

In the present study, we found that cuprizone feeding for 5 weeks significantly impaired short-term spatial working memory, as evidenced by the Y-maze spontaneous alteration task. The percentage of spontaneous alterations in the CPZ group was significantly lower than that of the control group, indicating memory deficits. The BFZ (blank formulation) and CFZ (curcumin alone formulation) treatment groups showed similar results to the CPZ group, suggesting that these formulations did not provide protective benefits against cuprizone toxicity. In contrast, the PFZ group (curcumin + piperine formulation) exhibited a slight increase in spontaneous alterations, although this increase was not statistically significant compared to the CPZ group. After the withdrawal of cuprizone and continuation of treatment for an additional 2 weeks, the percentage of spontaneous alterations increased across all groups when compared to the levels observed at the end of week 5.

In the NADT, mice from the CPZ group spent less time in the novel arm compared to the control group, indicating impaired memory function. However, mice from the treatment groups, particularly BFZ and PFZ, spent significantly more time in the novel arm compared to the control and CPZ groups, as shown by the NADT results at both week 5 and week 7. Interestingly, the CFZ group, which received curcumin alone, did not exhibit any significant improvement, spending a similar amount of time in the novel arm as the CPZ group at week 5. By week 7, mice in the CFZ group spent significantly less time in the novel arm compared to the CPZ group (*p* ≤ 0.05), suggesting that curcumin alone was not as effective as the combination formulation with piperine.

In the NORT, which assesses learning and memory in mice, we observed a significant reduction in the sniffing frequency for the novel object in the CPZ group compared to the familiar object at the end of week 5. However, the treatment groups showed a better preference for the novel object, with significantly higher sniffing frequencies compared to the CPZ group. This pattern suggests that the nanoformulations provided a protective effect against cuprizone-induced memory impairment. These findings align with previous studies conducted in our lab and by other researchers (35). Overall, after 5 weeks of cuprizone treatment, our results demonstrate significant impairments in spatial and recognition memory in the CPZ group. Previous studies have linked cuprizone-induced spatial memory deficits to demyelination, microglial activation, and elevated levels of pro-inflammatory cytokines in various brain regions (12). Additionally, enhanced inflammatory responses from microglia and astrocytes have been correlated with recognition memory impairments in mice exposed to cuprizone (36).

In the present study, an unexpected outcome was observed in the Novel Object Recognition Test, where the Novelty Preference Index in all groups was below 50% at week 5, indicating that the animals spent more time exploring the familiar object than the novel one. This reversal of preference is atypical, as rodents normally exhibit a spontaneous inclination toward novelty (37). Several factors may explain this phenomenon. First, cuprizone-induced neurotoxicity can impair recognition memory early in the course of demyelination and hippocampal dysfunction, leading to reduced discrimination between novel and familiar stimuli (12). Second, preference for the familiar object may also reflect increased anxiety or altered exploratory behavior, as rodents under stress or with hippocampal-prefrontal circuit impairment may avoid novel stimuli (34). Methodological influences, such as object salience, odor cues, or habituation differences, could further bias animals toward familiar exploration. However, it was observed that in Week 5, novelty preference in groups like in CPZ, BFZ and CFZ was below the novelty preference level observed in CNT and PFZ group. The higher novelty preference in PFZ group is indicative of beneficial effects of curcumin-piperine combination.

By week 7, animals in the control (CNT) and cuprizone (CFZ) groups exhibited nearly equal exploration of both objects, suggesting an absence of novelty recognition. This may reflect habituation to the testing environment or the development of recognition memory fatigue due to repeated exposures across weeks (38). In contrast, the treatment groups showed a clear preference for the novel object, suggesting that curcumin–piperine nanoparticles and *Z. rhetsa* oil helped restore recognition-based memory. This pattern highlights that while untreated animals either failed to discriminate or exhibited reduced motivation to explore novel stimuli, the therapeutic interventions mitigated these deficits and reinstated novelty preference.

Taken together, these results suggest that the lack of novelty preference at week 5 may represent a combination of cuprizone-induced memory dysfunction and heightened stress responses, whereas the persistent absence of preference in the control and CFZ groups at week 7 points toward recognition fatigue or reduced cognitive flexibility. Importantly, the restoration of novelty preference in treated groups suggests the neuroprotective and cognitive-enhancing effects of the interventions. Future studies with larger sample sizes and modified NORT protocols (e.g., varying inter-trial intervals, object sets, and environmental enrichment) may help disentangle biological deficits from task-related influences.

### 4.2 Biochemical studies

#### 4.2.1 Oxidative stress markers

The present study evaluated the biochemical impact of CPZ-induced neurotoxicity and assessed the therapeutic efficacy of various nanoformulations, including curcumin (CFZ), curcumin with piperine (PFZ), and a blank formulation (BFZ), by monitoring oxidative stress biomarkers and pro-inflammatory mediators over a 7-weeks period. Cuprizone-induced neurotoxicity was evident from a marked reduction in key antioxidant enzymes such as CAT, SOD, GSH, and GPX at Week 5. The suppression of CAT activity in the CPZ group aligns with existing studies indicating CPZ-mediated mitochondrial dysfunction and glial activation as critical sources of ROS, which in turn reduce the levels of intrinsic antioxidants like CAT (39). Among the treated groups, BFZ exhibited higher CAT levels at Week 7 compared to CPZ and control, suggesting possible endogenous compensation or minor antioxidant activity inherent to the formulation’s excipients. The CFZ group demonstrated a substantial recovery in CAT activity by Week 7, reinforcing the notion that curcumin facilitates ROS scavenging and supports antioxidant regeneration pathways (40). Surprisingly, PFZ-treated animals exhibited a lower CAT recovery than CFZ, despite the anticipated bio-enhancing role of piperine. This may be attributed to saturation effects or altered cellular metabolism upon prolonged co-administration. SOD activity, a primary defense against superoxide radicals, showed significant elevation in all groups by Week 7, with the CPZ group exhibiting a 252.7% increase. While this surge could indicate a reactive upregulation to persistent oxidative damage, the therapeutic groups especially CFZ exhibited a more controlled enhancement in SOD levels, implying functional restoration rather than compensatory overactivation. Similar antioxidant responses to curcumin and its formulations have been reported in models of neurodegeneration (41). GSH levels mirrored oxidative stress progression, with a notable depletion in the CPZ group. Interestingly, the PFZ group showed the highest GSH concentration at Week 5, indicating an initial robust antioxidant response, likely due to piperine-mediated enhancement of curcumin’s cellular uptake (42). However, by Week 7, GSH levels in the PFZ group dropped below baseline, suggesting a possible feedback inhibition or depletion of GSH biosynthetic precursors due to sustained metabolic demand. In contrast, CFZ and BFZ sustained moderately elevated GSH levels over time, suggesting better maintenance of redox homeostasis. The observed reduction in SOD (50.24%) and GSH (47.8%) levels in the control group between weeks 5 and 7 likely reflects an age-associated decline in antioxidant defense mechanisms. Previous studies have documented similar trends in age-related reduction of enzymatic antioxidant levels in mice as well as in humans even over short durations. There are many possible mechanisms that can lead to decreased SOD activity with age such as glycation, increased ROS generation, decreased mRNA expression, and deficits of zinc and copper ions (43). The GPX profile echoed similar patterns: an initial suppression in the CPZ group, with dramatic upregulation in PFZ (Week 5), followed by stable or slightly reduced levels in treated groups by Week 7. These results are consistent with studies highlighting the capacity of curcumin to activate Nrf2 and related antioxidant pathways (44). The PFZ group’s decline could reflect a transient overcompensation phase followed by a regulatory downshift.

#### 4.2.2 Neuroinflammatory markers

Astrocytic activation, indicated by GFAP expression, surged in all CPZ-exposed groups at Week 5. Surprisingly, nanoformulation-treated groups showed even higher GFAP levels than CPZ alone, possibly due to the delivery vehicle inducing transient irritation or an immune response (45). By Week 7, however, PFZ and CFZ groups displayed significant GFAP reductions, with PFZ nearing baseline levels, highlighting the therapeutic potential of curcumin-based nanoformulations in mitigating glial activation and restoring homeostasis. MCP-1 is a and MIP-1 levels, two critical chemokines implicated in monocyte recruitment and microglial activation, followed the expected trajectory elevated in CPZ, partially reversed in BFZ and CFZ, and significantly suppressed in PFZ compared to the CPZ group. Despite PFZ treatment, MCP-1 levels increased in week 7, possibly reflecting a secondary immune rebound after CPZ withdrawal. While PFZ attenuated acute inflammatory responses, this delayed elevation may signify a chronic neuroinflammatory cascade. These findings support earlier studies suggesting that curcumin, particularly when bioavailability is enhanced, suppresses NF-κB-mediated chemokine production and thereby reduces leukocyte infiltration in neuroinflammatory conditions (46). CCL-5 expression showed similar trends. The PFZ and CFZ groups demonstrated normalization of CCL-5 by Week 7, while BFZ maintained intermediate levels. The reduction in CCL-5 aligns with studies linking curcumin to the suppression of CCR5-mediated inflammatory signaling in the central nervous system (47).

The observed changes in hippocampal MBP levels provide further evidence that curcumin–piperine nanoformulation exerts potent protective effects against cuprizone-induced demyelination. The marked reduction in MBP in the CPZ group reflects oligodendrocyte damage and progressive myelin loss, consistent with the established neurotoxic profile of cuprizone (7, 48). Partial recovery in the BFZ and CFZ groups suggests that both blank/vehicle and curcumin nanoformulations confer some degree of protection, likely through attenuation of oxidative stress and modulation of inflammatory cascades. However, the most striking effect was seen in the PFZ group, which not only preserved MBP in Week 5 but also enhanced MBP levels by Week 7, surpassing control values. This suggests that piperine synergistically augments the bioavailability and neuroprotective actions of curcumin, stabilizing oligodendrocyte function and promoting remyelination. The robust increase in MBP over time highlights the dual antioxidant and anti-inflammatory mechanisms of the curcumin–piperine complex, which together counteract cuprizone-induced myelin damage and support structural integrity of the hippocampus (13).

#### 4.2.3 Biochemical results corroborate behavioral findings

The biochemical changes in the present study correlated closely with alterations in cognitive and behavioral performance, providing translational insight into the mechanistic underpinnings of neurodegenerative recovery. For example, at Week 5, mice subjected to CPZ feeding exhibited a significant decline in antioxidant enzymes (CAT, SOD, GPX, and GSH), accompanied by elevated levels of neuroinflammatory markers such as GFAP, MCP-1, MIP-1, and CCL-5. This biochemical disruption was mirrored in behavioral impairments. Spontaneous alternation and novel arm discrimination tests revealed deficits in spatial working memory, with CPZ-treated mice displaying significantly lower performance compared to controls. The NORT further demonstrated reduced preference for novel stimuli, suggesting impairments in recognition memory and cognitive flexibility. These deficits align with previous reports demonstrating that CPZ induces demyelination and hippocampal oxidative damage, leading to impaired short-term memory and executive function (39).

Among the treatments, PFZ emerged as the most effective in restoring both biochemical and behavioral deficits. Biochemically, PFZ treatment produced highest GPX and GSH levels at Week 5. This treatment also results in significant reduction in inflammatory cytokines (GFAP, MCP-1, MIP-1, CCL-5) by Week 7. It also found to sustain moderate antioxidant activity (e.g., 39.5% increase in CAT from Week 5 to Week 7). Behaviorally, PFZ-treated mice showed significant improvement in spontaneous alternation by Week 7, recovering to control-like levels, indicating restored hippocampal function and working memory. Cognition results indicated a normalization in novel arm discrimination, suggesting enhanced spatial learning and memory consolidation. In NORT, PFZ mice demonstrated significantly improved novelty preference, aligning with their superior biochemical recovery in antioxidant and anti-inflammatory parameters. The enhanced performance of PFZ is likely attributed to piperine’s role in increasing curcumin’s bioavailability by inhibiting glucuronidation and enhancing gastrointestinal absorption, thus amplifying curcumin’s neuroprotective effects through ROS scavenging and anti-inflammatory modulation (49).

Curcumin-only treatment (CFZ) showed moderate improvement in biochemical markers (e.g., 260% increase in CAT, 93.4% increase in SOD by Week 7), but less behavioral improvement compared to PFZ. In the novel arm discrimination test, CFZ mice spent significantly less time in the novel arm at Week 7 than even CPZ and PFZ groups, suggesting impaired spatial novelty recognition, possibly due to transient or regionally limited neuroprotection. Despite elevated GSH and GPX levels in CFZ, the behavioral outcomes were not commensurate, possibly due to incomplete mitigation of neuroinflammatory processes, as indicated by relatively higher GFAP and MCP-1 levels compared to PFZ. BFZ (blank formulation) surprisingly conferred mild biochemical and behavioral benefits, possibly due to inert vehicle effects. BFZ-treated mice showed Higher SOD and GSH levels than CPZ, suggesting some oxidative protection. Improved novel arm discrimination and NORT performance compared to CPZ, though not matching PFZ, implying that the formulation matrix may provide some support to cellular homeostasis.

Astrocytic activation, reflected by GFAP expression, was high across all CPZ-exposed groups at Week 5 but significantly attenuated in PFZ and CFZ groups by Week 7. The strong GFAP reduction in PFZ mice (from 51.33 to 11.53 pg/mg) correlated with their improved performance in all behavioral assays, emphasizing the link between neuroinflammation and cognitive impairment. Similarly, suppression of MCP-1, MIP-1, and CCL-5 in PFZ-treated animals supports curcumin’s role in modulating microglial activity and reducing cytokine-induced synaptic disruption (50). This neuroimmune resolution appears critical for restoring behavioral performance. The PFZ group’s low GFAP and chemokine levels paralleled high SOD and GPX activity, a biochemical environment conducive to neuronal plasticity and cognitive recovery (51).

The preservation of MBP levels observed particularly in the PFZ group strongly correlates with the improved cognitive performance demonstrated in behavioral assays. Since myelin integrity in the hippocampus is essential for efficient synaptic transmission and neuronal connectivity, the loss of MBP in CPZ-treated animals likely contributed to the cognitive impairment observed (52). Partial restoration of MBP in BFZ and CFZ groups may explain their modest improvements in behavioral performance, whereas the marked enhancement in MBP in the PFZ group is consistent with its superior effects on learning and memory. These findings suggest that the curcumin–piperine nanoformulation not only mitigates oxidative and inflammatory damage but also preserves structural substrates of cognition, thereby linking molecular protection of myelin with functional recovery in cognitive domains.

The close alignment between biochemical and behavioral outcomes in this study emphasize the utility of combinatorial nanoformulations in managing neuroinflammatory demyelinating conditions. Specifically, PFZ’s efficacy highlights a promising translational candidate for disorders like multiple sclerosis, where oxidative stress and cognitive decline are central features. Moreover, the temporal dynamics observed early biochemical restoration (Week 5) followed by behavioral improvement (Week 7) support a model in which redox and immune homeostasis precede functional recovery, offering a framework for future mechanistic studies and therapeutic interventions.

### 4.3 Histological studies

We focused our histological analyses on the dentate gyrus (DG) because this hippocampal subregion is particularly vulnerable to cuprizone-induced demyelination, oxidative stress, and neuroinflammatory responses (7, 8). The DG plays a pivotal role in hippocampal neurogenesis, pattern separation, and the encoding of new memories, making it highly relevant to the behavioral domains assessed in our study, such as spatial working memory and recognition memory (53). Previous studies have shown that structural and cellular alterations in the DG closely correlate with deficits observed in the Y-maze and NORT, further supporting its selection as a target region for analysis (54). Thus, restricting histological evaluation to the DG allowed us to directly link neuropathological changes with functional outcomes, while maintaining methodological focus.

The histological findings corroborate the biochemical and behavioral results, highlighting the structural damage induced by cuprizone and the neuroprotective efficacy of the nanoformulations. At Week 5, CPZ-treated mice exhibited marked degeneration of the dentate gyrus with vacuolization and neuronal death. In contrast, PFZ-treated mice showed near-normal architecture, supporting the superior neuroprotective potential of the curcumin-piperine combination. The BFZ and CFZ groups showed moderate preservation, suggesting partial efficacy. By Week 7, there was clear histological recovery in all groups, most notably in the PFZ group, where normal granule layer structure was largely restored. These observations suggest that the nanoformulated curcumin, especially when paired with piperine, helps in preserving hippocampal architecture possibly by reducing oxidative stress and neuroinflammation, and enhancing endogenous repair mechanisms. The alignment of histological, biochemical, and behavioral improvements in PFZ-treated animals strongly supports its therapeutic potential in CPZ-induced neurotoxicity. While H&E staining provided morphological evidence, the inclusion of specific markers like Fluoro-Jade, Iba1, or TUNEL assays would enhance mechanistic insight, which we plan to address in future studies.

In line with the ARRIVE 2.0 guidelines, we have ensured comprehensive reporting of study design, randomization, group sizes, attrition, outcome measures, and statistical analyses. Explicit adherence to these guidelines enhances the reproducibility, ethical transparency, and interpretability of our findings.

## 5 Conclusion

This study highlights the detrimental impact of cuprizone on neurocognitive function, emphasizing its role in inducing oxidative stress and neuroinflammation. The findings demonstrate that prophylactic treatment with curcumin and piperine nanoformulations mitigates these effects by restoring antioxidant enzyme levels and reducing inflammatory markers. Behavioral and histological analyses further confirmed the protective role of these nanoformulations, particularly the curcumin-piperine combination, in preserving cognitive function. While curcumin alone showed moderate benefits, its combination with piperine exhibited enhanced efficacy, likely due to improved bioavailability and synergistic anti-inflammatory properties. Overall, the results support the therapeutic potential of curcumin and piperine nanoformulations in managing neuroinflammatory disorders and can be used as an adjuvant therapy along with standard approved treatments available. Further studies are warranted to elucidate their precise mechanisms of action and optimize formulations for clinical applications.

### 5.1 Limitations

This study has several limitations that should be considered when interpreting the findings. Retesting within a short 2-week interval may have introduced long-lasting memory traces that influenced performance outcomes. This limitation highlights the need for extended retest intervals or separate cohorts to reduce the confounding effects of prior exposure in future behavioral studies. Using the same cohort for multiple behavioral tasks increases the risk of cross-test interference and may reduce the specificity of individual assessments. Future work will address this by employing distinct animal groups for different behavioral paradigms. The absence of novelty preference in control groups at both week 5 and week 7 suggests methodological shortcomings, likely related to object selection, test intervals, or insufficient counterbalancing. To overcome this, future experiments will adopt longer retention intervals (5–10 min) and utilize counterbalanced, commercially available objects to ensure more reliable novelty discrimination. Additionally, the precise mechanism underlying the mild protective effects observed in the BFZ group remains unclear, warranting further histopathological and molecular analyses. The transient antioxidant recovery in PFZ-treated animals also indicates complex regulatory feedback requiring deeper investigation, and the absence of confirmatory neuropathological assays such as TUNEL or Fluoro-Jade staining limited assessment of neuronal degeneration.

### 5.2 Implications

Despite these limitations, our findings provide meaningful insights into the therapeutic potential of curcumin nanoformulations. Notably, PFZ exhibited early but transient antioxidant responses, whereas CFZ produced more consistent and sustained biochemical improvements. These results suggest that formulation strategy plays a critical role in optimizing therapeutic efficacy. Moreover, the observation of partial protective effects in the BFZ group underscores the need to carefully consider vehicle contributions in nanoformulation studies. Collectively, these findings support the broader hypothesis that curcumin-based nanoformulations, particularly when combined with piperine, may mitigate oxidative stress and neuroinflammation during demyelination.

## Data Availability

The original contributions presented in this study are included in this article/supplementary material, further inquiries can be directed to the corresponding author.

## Footnotes

1
https://www.equator-network.org/
